# Use of the Decipher genomic classifier among men with prostate cancer in the United States

**DOI:** 10.1093/jncics/pkad052

**Published:** 2023-08-01

**Authors:** Nicholas G Zaorsky, James A Proudfoot, Angela Y Jia, Raed Zuhour, Randy Vince Jr, Yang Liu, Xin Zhao, Jim Hu, Nicola C Schussler, Jennifer L Stevens, Suzanne Bentler, Rosemary D Cress, Jennifer A Doherty, Eric B Durbin, Susan Gershman, Iona Cheng, Lou Gonsalves, Brenda Y Hernandez, Lihua Liu, Bożena M Morawski, Maria Schymura, Stephen M Schwartz, Kevin C Ward, Charles Wiggins, Xiao-Cheng Wu, Jonathan E Shoag, Lee Ponsky, Alan Dal Pra, Edward M Schaeffer, Ashley E Ross, Yilun Sun, Elai Davicioni, Valentina Petkov, Daniel E Spratt

**Affiliations:** Department of Radiation Oncology, University Hospitals Seidman Cancer Center, Cleveland, OH, USA; Department of Population and Quantitative Health Sciences, Case Western Reserve School of Medicine, Case Western Reserve University, Cleveland, OH, USA; Veracyte, Inc, South San Francisco, CA, USA; Department of Radiation Oncology, University Hospitals Seidman Cancer Center, Cleveland, OH, USA; Department of Population and Quantitative Health Sciences, Case Western Reserve School of Medicine, Case Western Reserve University, Cleveland, OH, USA; Department of Radiation Oncology, University Hospitals Seidman Cancer Center, Cleveland, OH, USA; Department of Population and Quantitative Health Sciences, Case Western Reserve School of Medicine, Case Western Reserve University, Cleveland, OH, USA; Department of Radiation Oncology, University Hospitals Seidman Cancer Center, Cleveland, OH, USA; Department of Population and Quantitative Health Sciences, Case Western Reserve School of Medicine, Case Western Reserve University, Cleveland, OH, USA; Veracyte, Inc, South San Francisco, CA, USA; Veracyte, Inc, South San Francisco, CA, USA; Department of Urology, Weil Cornell Medicine, New York, NY, USA; Information Management Systems, Inc, Calverton, MD, USA; Information Management Systems, Inc, Calverton, MD, USA; Iowa Cancer Registry, The University of Iowa, IA, USA; Public Health Institute, Cancer Registry of Greater California, Sacramento, CA, USA; Huntsman Cancer Institute, University of Utah, Salt Lake City, UT, USA; Department of Population Health Sciences, University of Utah, Salt Lake City, UT, USA; Cancer Research Informatics Shared Resource Facility, Markey Cancer Center, Kentucky Cancer Registry, University of Kentucky, Lexington, KY, USA; Massachusetts Cancer Registry, Boston, MA, USA; Department of Epidemiology and Biostatistics, University of California, San Francisco, CA, USA; Connecticut Tumor Registry, Connecticut Department of Public Health, Hartford, CT, USA; University of Hawaii Cancer Center, HI, USA; Department of Population and Public Health Sciences, Keck School of Medicine, University of Southern California, Los Angeles, CA, USA; Cancer Data Registry of Idaho, Boise, ID, USA; School of Public Health Epidemiology & Biostatistics, University at Albany, State University of New York, NY, USA; Division of Public Health Sciences, Fred Hutchinson Cancer Center, Seattle, WA, USA; Rollins School of Public Health, Emory University, Atlanta, GA, USA; Department of Internal Medicine, University of NM, Albuquerque, NM, USA; Department of Epidemiology, School of Medicine, Louisiana State University, New Orleans, LA, USA; Department of Radiation Oncology, University Hospitals Seidman Cancer Center, Cleveland, OH, USA; Department of Population and Quantitative Health Sciences, Case Western Reserve School of Medicine, Case Western Reserve University, Cleveland, OH, USA; Department of Radiation Oncology, University Hospitals Seidman Cancer Center, Cleveland, OH, USA; Department of Population and Quantitative Health Sciences, Case Western Reserve School of Medicine, Case Western Reserve University, Cleveland, OH, USA; Department of Radiation Oncology, University of Miami, Miami, FL, USA; Department of Urology, Northwestern University, Chicago, IL, USA; Department of Urology, Northwestern University, Chicago, IL, USA; Department of Population and Quantitative Health Sciences, Case Western Reserve School of Medicine, Case Western Reserve University, Cleveland, OH, USA; Veracyte, Inc, South San Francisco, CA, USA; Surveillance Research Program, National Cancer Institute, Bethesda, MD, USA; Department of Radiation Oncology, University Hospitals Seidman Cancer Center, Cleveland, OH, USA; Department of Population and Quantitative Health Sciences, Case Western Reserve School of Medicine, Case Western Reserve University, Cleveland, OH, USA

## Abstract

**Background:**

Management of localized or recurrent prostate cancer since the 1990s has been based on risk stratification using clinicopathological variables, including Gleason score, T stage (based on digital rectal exam), and prostate-specific antigen (PSA). In this study a novel prognostic test, the Decipher Prostate Genomic Classifier (GC), was used to stratify risk of prostate cancer progression in a US national database of men with prostate cancer.

**Methods:**

Records of prostate cancer cases from participating SEER (Surveillance, Epidemiology, and End Results) program registries, diagnosed during the period from 2010 through 2018, were linked to records of testing with the GC prognostic test. Multivariable analysis was used to quantify the association between GC scores or risk groups and use of definitive local therapy after diagnosis in the GC biopsy-tested cohort and postoperative radiotherapy in the GC-tested cohort as well as adverse pathological findings after prostatectomy.

**Results:**

A total of 572 545 patients were included in the analysis, of whom 8927 patients underwent GC testing. GC biopsy-tested patients were more likely to undergo active active surveillance or watchful waiting than untested patients (odds ratio [OR] =2.21, 95% confidence interval [CI] = 2.04 to 2.38, *P* < .001). The highest use of active surveillance or watchful waiting was for patients with a low-risk GC classification (41%) compared with those with an intermediate- (27%) or high-risk (11%) GC classification (*P* < .001). Among National Comprehensive Cancer Network patients with low and favorable-intermediate risk, higher GC risk class was associated with greater use of local therapy (OR = 4.79, 95% CI = 3.51 to 6.55, *P* < .001). Within this subset of patients who were subsequently treated with prostatectomy, high GC risk was associated with harboring adverse pathological findings (OR = 2.94, 95% CI = 1.38 to 6.27, *P* = .005). Use of radiation after prostatectomy was statistically significantly associated with higher GC risk groups (OR = 2.69, 95% CI = 1.89 to 3.84).

**Conclusions:**

There is a strong association between use of the biopsy GC test and likelihood of conservative management. Higher genomic classifier scores are associated with higher rates of adverse pathology at time of surgery and greater use of postoperative radiotherapy.

In this study the Decipher Prostate Genomic Classifier (GC) was used to analyze a US national database of men with prostate cancer. Use of the GC was associated with conservative management (ie, active surveillance). Among men who had high-risk GC scores and then had surgery, there was a 3-fold higher chance of having worrisome findings in surgical specimens.

Since the 1990s, the management of localized or recurrent prostate cancer has been based on risk stratification using clinicopathological variables, including Gleason score, T stage (based on digital rectal examination), and prostate specific antigen (PSA). Prognostic discrimination is improved by incorporation of these variables together into multivariable models. Other variables, like PSA density, extent of biopsy core involvement, and Gleason pattern, may help in prognostication (1). The performances of most published models are modest and there is a clinical need for biomarkers to improve prognostication and prediction for personalized treatment decision making.

The GC is one such method developed to meet this need (2). The GC has been validated in randomized clinical trials in men with newly diagnosed intermediate-risk, high-risk, postprostatectomy, biochemically recurrent, nonmetastatic castration resistant, and metastatic hormone sensitive prostate cancers (2-6). The GC has been shown to consistently improve prognostic discrimination, add independent prognostic information above and beyond clinicopathological variables, and change treatment decisions.

In 2019, The San Francisco Consensus Statement on prostate cancer biomarkers (7) was published with the aim of helping to guide future research on biomarker use. This Statement focused on 6 key points for adopting novel biomarkers: (a) use in a setting where a change in management is possible from the biomarker result; (b) ensure that prognostic biomarkers are independently predictive when incorporated into multivariable models; (c) ensure that prognostic biomarkers improve discrimination for clinically meaningful endpoints such as distant metastasis and cause-specific and overall survival; (d) assess in high-quality studies [eg, using Simon criteria (8)]; (e) assess in diverse patient populations; and (f) ensure that test results are conveyed to patients that may affect shared decision-making with their physician regarding changing management of their disease (9). While the GC meets all 6 of these criteria, to our knowledge its use has not yet been assessed using a US population-based data source. The purpose of this report is to characterize GC usage and its association with treatment decisions for men diagnosed with prostate cancer in the United States.

## Methods

The Surveillance, Epidemiology, and End Results (SEER) program of the National Cancer Institute collects cancer incidence information, including patient demographic data, clinical characteristics of the cancer (primary site, histology, grade, behavior, stage, extent of disease), first course of treatment, and follow-up information. At the time of this study, SEER covered approximately 34.6% of the US population and captured at least 98% of incident cancer cases within the catchment areas (10). In 2021, records from the Decipher GC and SEER cancer registries [SEER-18 (11)] were linked for the first time.

Records from participating SEER Program registries for prostate cancer patients diagnosed during the period from 2010 through 2018 were linked to records of GC testing conducted between 2013 and 2020 with Decipher GC (Veracyte, San Diego, CA). Linkage was tested and validated by using personally identifiable information with cancer records within each registry by a third-party honest broker (IMS, Rockville, MD). The SEER registries collect data on sex, age at diagnosis, race and ethnicity, marital status, census tract socioeconomic status, and year of diagnosis. Data for which SEER does not code specific comorbidities include performance status, radiotherapy details (eg, dose, volume), and systemic therapy details (eg, agent, duration, dose). All treatment information collected by the SEER registries came from the medical records recorded by the managing physician(s) in the treatment plan and administered within the first 6 months after diagnosis (before disease progression or recurrence) and are defined as “First Course of Treatment (or Therapy)”.

Clinical and pathological variables within SEER were used in all analyses, and any overlapping data found in the Decipher GC database were only used for linkage. The only data merged from the Decipher GC database were the continuous and categorial GC score and year the test was conducted. Very low and low National Comprehensive Cancer Network (NCCN) risk groups (12) were combined because PSA density was unavailable within SEER. Patients who were otherwise intermediate risk but did not have available data on percentage of positive cores were categorized as intermediate (not otherwise specified [NOS]).

Summary statistics for continuous and categorical demographic, clinical, and pathological characteristics were reported as medians (IQRs) and counts (percentages), respectively. GC scores (range 0-1) and GC risk groups (locked commercial cut points, defined as low [<0.45], intermediate [0.45-0.60], and high risk [>0.6]) were used for continuous and categorical analyses (13). Neighborhood socioeconomic status was defined using the Yost index, which comprises multiple domains related to socioeconomic status (14). The statistical significance of differences in clinicopathological variables across GC risk groups was determined by Kruskal-Wallis tests for continuous data and χ^2^ tests for categorical data. Standardized mean differences (SMD) are reported between GC tested and untested populations.

Multivariable logistic regression was used to quantify the association between GC scores or risk groups and use of definitive local therapy (radical prostatectomy [RP] or radiation therapy [RT]) after diagnosis in the GC biopsy-tested cohort and radiotherapy in the GC prostatectomy-tested cohort. Variables in the model included GC score (continuous variable), age (continuous, per 5 years), diagnosis year (pre-2015, 2016, 2017, 2018), race (Asian or Pacific Islander, Black, Hispanic [all races], White, other), marital status (single, married, separated, unknown), census tract socioeconomic quintile (1 through 5), PSA (continuous, log2 transformed), percentage of positive cores, clinical grade group, and T stage. Similar models were fit for the occurrence of adverse pathology (15) (eg, pathological stage T3/4, lymph node invasion [LNI], or pathological grade group [pGG] 4 to 5) among GC RP-tested cohorts. Patients with missing covariate data were excluded from multivariable models. A Bonferroni-Holm adjustment was applied to GC *P* values across models where both continuous and categorical GC effects were estimated. All analyses were performed using R version 3.6.3 (R Foundation for Statistical Computing, Vienna, Austria).

**Figure 1.**
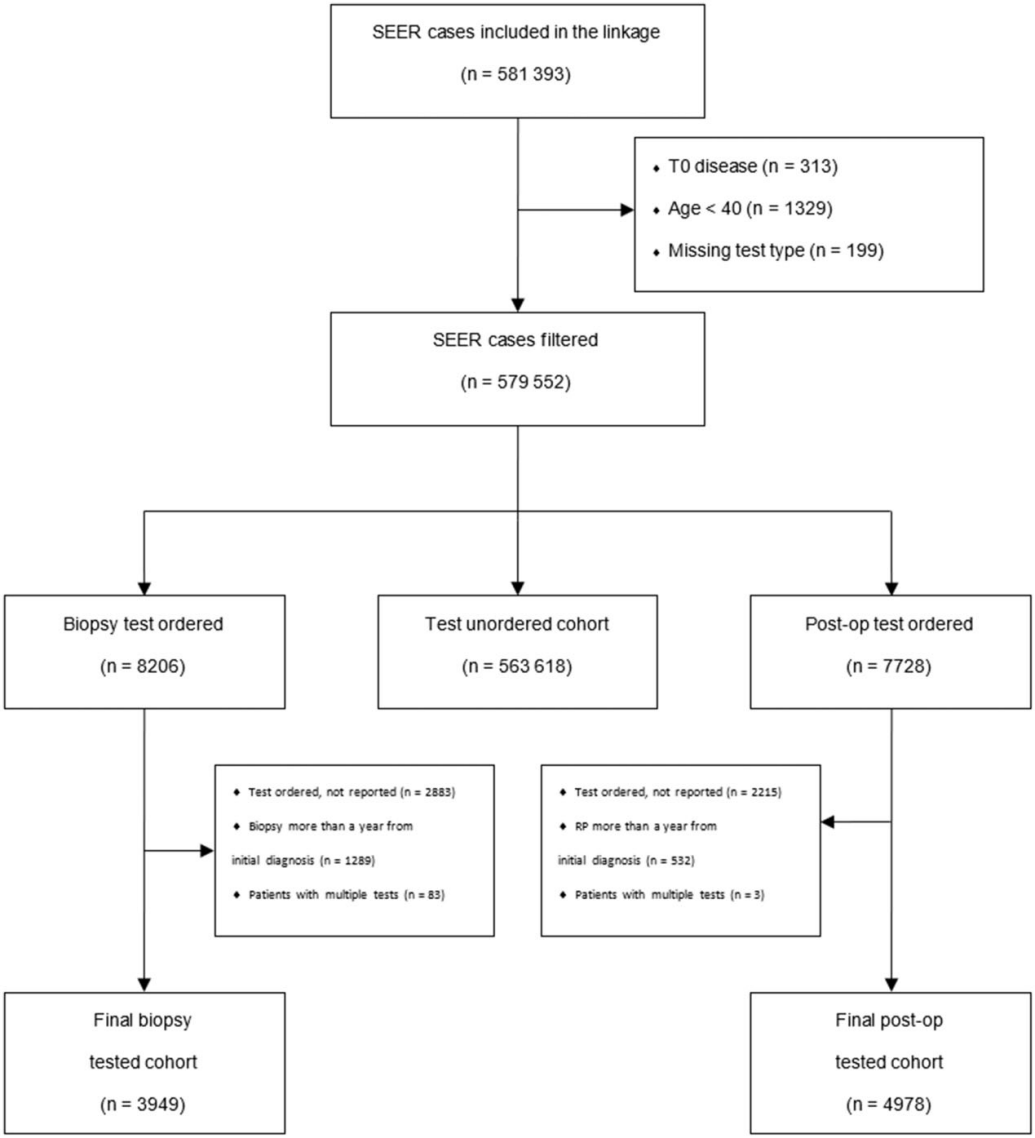
CONSORT diagram. SEER = Surveillance, Epidemiology, and End Results.

A total of 581 393 incident prostate cancer patients were diagnosed between 2010 and 2018 and included in the linkage. We excluded patients who were younger than 40 years or who had “T0” disease (Figure 1). Among the patients with GC testing ordered, we excluded those who had tumors for which the tissue sample used for GC was more than a year from their initial diagnosis, had repeated tests ordered, or had a test ordered but did not have a reported GC (either due to failing quality control such as insufficient tumor for testing or test cancellation by patient or provider). A total of 572 545 patients (563 618 unordered + 3949 GC biopsy + 4978 GC RP) were included in the analysis, of which 8927 patients underwent GC testing.

## Results

### Associations and trends with GC testing

**Figure 2.**
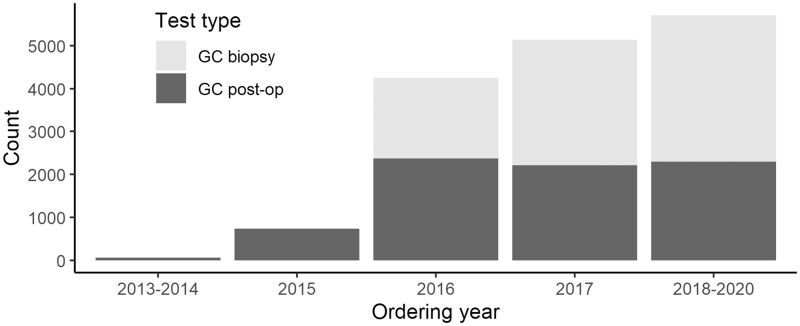
Test-ordering volume over time. GC = genomic classifier.

**Figure 3.**
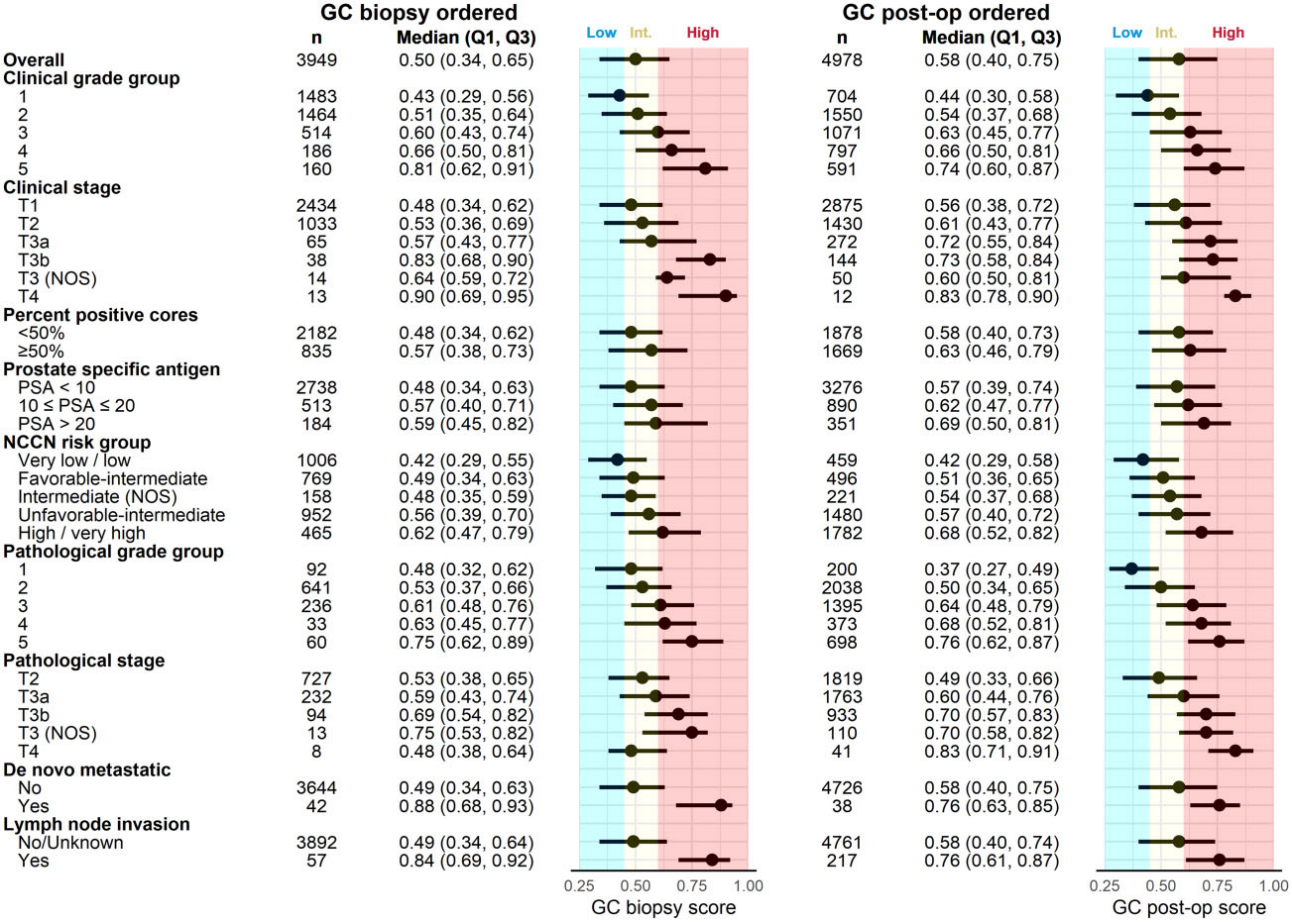
Forest plot of GC score distributions by clinical and pathological characteristics for GC biopsy and GC prostatectomy scores, respectively. Data are presented as median (first quartile [Q1], third quartile [Q3]), with low, intermediate, and high regions defined by previously locked commercial cut points. GC = genomic classifier.

Ordering for GC prostatectomy tests was made available in a limited capacity in 2013, while GC biopsy tests were first offered in 2016 with coverage approval only for low-risk prostate cancer (Figure 2). The GC-tested cohort was younger, more likely to be non-Hispanic White, married, and of higher census tract socioeconomic status compared with the untested cohort (Supplementary Table 1, Supplementary Figure 1, available online). Higher GC scores were associated with higher clinical or pathological grade and stage (Figure 3), higher percentage of positive cores and PSA, higher likelihood of de novo metastasis and lymph node invasion, and higher NCCN risk group classification (all *P* < .001, Supplementary Figure 2, available online). Supplementary Table 2 (available online) shows clinical and pathological characteristics of patients stratified by test type and GC risk classification.

We conducted several analyses with different multivariable risk tools, including the international staging collaboration for cancer of the prostate (STAR-CAP) staging model (data available on request). In short, the breakdown among stages is similar and did not change the findings. We chose to display the data from NCCN risk groups because these are used by NCCN guidelines and are most familiar to physicians.

### Characteristics of the biopsy GC test at the time of diagnosis

Treatment information for the GC biopsy tested cohort stratified by GC risk classification is given in Supplementary Tables 3 (available online). On multivariable logistic regression, GC biopsy–tested patients were more likely to undergo active surveillance or watchful waiting (AS/WW) than untested patients (OR = 2.21; 95% CI = 2.05 to 2.39, *P* < .001, Supplementary Table 4, A, available online). This relationship remained when GC biopsy–tested patients were compared with untested patients, with initial diagnosis in 2016 and onward (ie, over the time period when GC biopsy testing was available) (OR = 2.05; 95% CI = 1.88 to 2.23, *P* < .001, Supplementary Table 4, B, available online).

**Figure 4.**
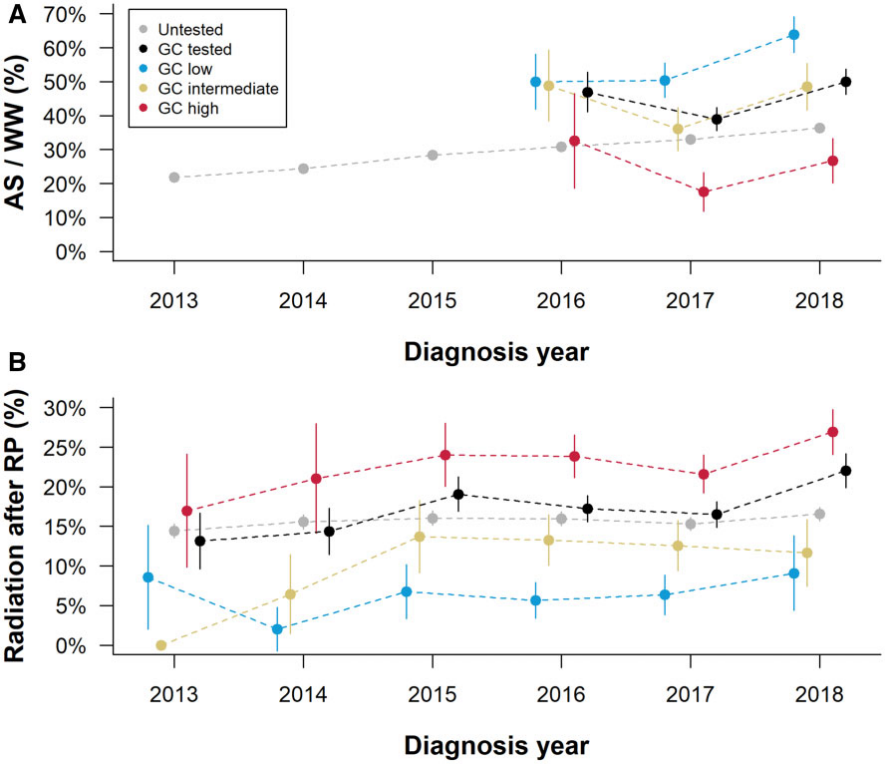
Rates of (A) active surveillance/watchful waiting among NCCN low and favorable-intermediate risk men and (B) radiation after radical prostatectomy among men with adverse pathological features (pT3/4, LN+, or pGG 4 to 5) in the GC untested and GC biopsy (A) and prostatectomy (B) tested cohorts. Vertical bars indicate 95% confidence intervals. GC = genomic classifier; LN+ = lymph-node positive; NCCN = National Comprehensive Cancer Network; pGG = pathological grade group.

Among patients with GC biopsy test results, AS/WW was highest for those with GC low-risk classification (41%) compared with those with intermediate (27%) or high (11%) GC risk (*P* < .001). AS/WW rates were higher in the GC biopsy tested cohort compared with the untested cohort when compared within NCCN strata both overall and when restricted to the time frame in which the GC biopsy test was offered (Table 1). There were consistently higher proportions of patients on AS/WW in the lower GC risk group classification and less AS/WW in higher GC risk patients since the test was introduced in 2016 (Table 1, Figure 4).

**Table 1.** Active surveillance/watchful waiting proportions within NCCN risk groups in the GC tested and untested cohorts^a^

	NCCN risk group					
	Very low and low	Favorable intermediate	Intermediate, not otherwise specified	Unfavorable intermediate	High and very high	Total
Untested	26.9% (33076/122857)	12.6% (6312/49965)	7.0% (1855/26577)	3.3% (3216/96327)	1.7% (1871/111753)	11.3% (46537/410717)
Untested (2016+)	42.2% (13652/32376)	17.1% (3003/17526)	8.9% (616/6956)	4.1% (1436/35276)	1.9% (784/42370)	14.5% (19544/134804)
GC biopsy tested	57.4% (577/1006)	29.1% (224/769)	13.3% (21/158)	9.8% (93/952)	3.2% (15/465)	27.7% (930/3353)
GC biopsy low risk	65.2% (356/546)	40.2% (130/323)	16.7% (11/66)	17.9% (57/318)	7.8% (8/103)	41.4% (562/1359)
GC biopsy intermediate risk	55.1% (158/287)	28.2% (64/227)	11.3% (6/53)	8.3% (20/241)	4.2% (5/118)	27.3% (253/926)
GC biopsy high risk	36.4% (63/173)	13.7% (30/219)	10.3% (4/39)	4.1% (16/393)	0.8% (2/244)	10.8% (115/1068)

^a^GC = genomic classifier; NCCN = National Comprehensive Cancer Network.

Among GC biopsy–tested patients, both prostatectomy and radiotherapy were increasingly more likely to be used in patients with higher GC risk; prostatectomy (22% of GC low, 30% of GC intermediate, and 39% of GC high risk, *P* < .001) and radiotherapy (16% of GC low, 24% of GC intermediate, and 35% of GC high risk, *P* < .001, Supplementary Table 3, available online). Among NCCN low- and favorable-intermediate patients with GC biopsy test results, multivariable logistic regression showed a statistically significant association between the use of local therapy (radiation and/or surgery) and both GC score (OR = 1.35, 95% CI = 1.26 to 1.44 per 0.1 increase, *P* < .001) and GC risk groups (OR = 1.89, 95% CI = 1.44 to 2.49, *P* < .001, and OR = 4.69, 95% CI = 3.44 to 6.39, *P* < .001) for intermediate and high vs low risk, respectively (Table 2).

**Table 2.** Multivariable logistic regression results for use of local therapy (radiation or surgery) in NCCN low/favorable-intermediate patients (n = 1482 patients/616 instances of local therapy with nonmissing covariate values)^a^

Variable	GC score model		GC risk group model	
	Multivariable OR (95% CI)	*P*	Multivariable OR (95% CI)	*P*
GC score	1.35 (1.26 to 1.44)	<.001^a^^,^^b^	—	—
GC risk group: intermediate vs low	—	—	1.89 (1.44 to 2.49)	<.001^a^^,^^b^
GC risk group: high vs low	—	—	4.69 (3.44 to 6.39)	<.001^a^^,^^b^
Age (per 5 years)	0.92 (0.84 to 1.00)	.04^b^	0.91 (0.84 to 0.99)	.03^b^
Diagnosis year 2016 vs ≤2015	0.66 (0.23 to 1.91)	.45	0.79 (0.28 to 2.27)	.67
Diagnosis year 2017 vs ≤2015	0.77 (0.28 to 2.17)	.62	0.90 (0.32 to 2.50)	.83
Diagnosis year 2018 vs ≤2015	0.50 (0.18 to 1.41)	.19	0.58 (0.21 to 1.64)	.31
Race NH Black vs NH White	1.01 (0.70 to 1.47)	.94	1.02 (0.70 to 1.47)	.93
Race NH Asian/Pacific Islander vs NH White	1.90 (0.94 to 3.83)	.07	1.90 (0.94 to 3.84)	.07
Race Hispanic (all races) vs NH White	1.12 (0.67 to 1.89)	.66	1.17 (0.69 to 1.97)	.56
Race NH other/unknown vs NH White	1.32 (0.58 to 2.98)	.51	1.36 (0.59 to 3.10)	.47
Marital status separated vs married	0.74 (0.48 to 1.14)	.17	0.72 (0.47 to 1.11)	.14
Marital status single vs married	0.67 (0.46 to 0.99)	.04^b^	0.67 (0.45 to 0.98)	.04^b^
Marital status unknown vs married	0.62 (0.36 to 1.07)	.08	0.61 (0.35 to 1.05)	.08
Census tract SES quintile 2 vs 1	0.83 (0.46 to 1.51)	.55	0.88 (0.48 to 1.60)	.68
Census tract SES quintile 3 vs 1	0.87 (0.50 to 1.52)	.62	0.85 (0.49 to 1.50)	.58
Census tract SES quintile 4 vs 1	0.62 (0.37 to 1.06)	.08	0.63 (0.37 to 1.07)	.09
Census tract SES quintile 5 vs 1	0.60 (0.36 to 1.01)	.05	0.60 (0.36 to 1.00)	.05^b^
Log_2_(PSA)	1.01 (0.83 to 1.23)	.91	0.99 (0.82 to 1.21)	.93
Percent positive cores (per 10%)	1.27 (1.18 to 1.37)	<.001^b^	1.28 (1.19 to 1.37)	<.001^b^
Clinical grade group 2 vs 1	4.55 (3.55 to 5.82)	<.001^b^	4.71 (3.67 to 6.04)	<.001^b^
Clinical stage T2 vs T1	1.12 (0.83 to 1.51)	.48	1.10 (0.81 to 1.48)	.55

a Bonferroni-Holm adjusted *P* values. CI = confidence interval; GC = genomic classifier; NCCN = National Comprehensive Cancer Network; NH = non-Hispanic; OR = odds ratio; PSA = prostate-specific antigen; SES = socioeconomic status.

b *P* < .05.

Within the subset of patients with low or favorable intermediate NCCN risk classification at the time of biopsy and who were subsequently treated with prostatectomy (n = 361), GC biopsy high-risk findings (>0.6) were associated with nearly 3 times the odds that the patient harbors adverse pathology (OR = 2.88, 95% CI = 1.35 to 6.17, Bonferroni-Holm adjusted *P* = .01) (Supplementary Table 5, available online). We performed additional analyses to modify the definition of adverse pathology (eg, to include only patients with pT3b disease or GG3). The results were similar (OR = 1.29, 95% CI = 1.09 to 1.52 per 0.1 change in GC) on adverse pathology defined as pT3+, pGG 4 to 5, or LN+ (OR = 1.35, 95% CI = 1.13 to 1.62 per a 0.1 change in GC on adverse pathology defined as pT3b+, pGG3-5, or LN+).

### Characteristics of the GC test after radical prostatectomy

Because GC was first available for prostatectomy patients, we examined the treatment patterns for men post-RP (Supplementary Table 6, available online). Results from multivariable logistic regression analyses regarding the use of radiation therapy after prostatectomy in men with adverse pathological features (ie, pT3/4, LN+, or pGG 4 to 5) and GC prostatectomy testing are summarized in Supplementary Table 7 (available online). Use of postoperative radiotherapy was statistically significantly associated with higher GC risk group (GC low risk as reference, GC intermediate risk [OR = 1.55, 95% CI = 1.04 to 2.31], GC high risk [OR = 2.69, 95% CI = 1.89 to 3.84]), as well as higher PSA, higher pathological stage, and lymph node–positive disease. In contrast, diagnosis year, race, and Yost index were not statistically significantly associated with receipt of radiotherapy postprostatectomy. Radiation after prostatectomy rates overall in the GC tested were not substantially increased as compared to GC untested patients with adverse pathologic features (Figure 4).

## Discussion

This study is the first, to our knowledge, to analyze the use of Decipher GC among men with prostate cancer in the United States with linkage to the 2013-2018 data from the cohort-based SEER Program registries. The results of this study validated the correlation of GC scores with NCCN risk groups, but with marked heterogeneity in GC scores within a given risk group (1). Patients with lower GC scores were statistically significantly associated with increased use of conservative management compared with untested patients (16). GC testing itself was not associated with higher rates of postprostatectomy radiotherapy (9). However, patients with high GC scores were more likely to receive postprostatectomy radiotherapy than untested patients. Finally, we found that patients with clinically low or favorable-intermediate risk with higher GC scores were statistically significantly more likely to have adverse pathology at time of prostatectomy (17).

These data show that patients with GC testing were more likely to choose active surveillance; this finding is a correlation and does not necessarily indicate clear causation. It's possible that physicians or patients who ordered or asked for the test were already more inclined to consider active surveillance and that the results confirmed, but did not actually influence, that decision. The only way to prove that the tests change behavior would be to do a randomized trial with and without the test, or a trial in which clinicians and patients recorded their initial treatment preference before the test results were known, followed by recording their preference after the results were obtained. G-MAJOR (NCT04396808), a randomized phase 3 trial conducted by the Michigan Urological Surgery Improvement Collaborative (MUSIC) aims to answer this question as well as to determine if the use of genomic testing leads to improved quality of life and oncological outcomes.

Although prior studies have demonstrated the comparable performance of the GC in Black or African American patients, the current study identified a disparity in who undergoes GC testing. This study showed that underrepresented racial and ethnic minority groups and those with lower census tract socioeconomic status were less likely to undergo GC testing (18).

The present study demonstrated an association with GC testing itself and the GC test results with clinically relevant observations on multivariable analyses. Given the consistent observation that GC use changes disease management, it is probable that future real-world studies will show a blunted effect of the prognostic value of the GC given patients with higher GC scores are treated more aggressively and those with lower GC scores are treated more conservatively. This would be a success and a desired byproduct of GC utilization, to cause change in management that reduces over- and undertreatment, ultimately improving patient outcomes.

The current results describe real-world associations, trends, and practice patterns and should not be viewed as causal given the study design and potential limitations. First, while we present comparisons between GC-tested and -untested cohorts on restricted timelines and adjust for both demographic and clinicopathological factors in all models, the observational nature of the SEER data may introduce potential unmeasured or unmeasurable confounding factors that, if accounted for, could alter the measured effect of GC. Second, multivariable regression analyses were limited to patients without missing covariate values, which may introduce bias. Third, given the rapid increase in use of the GC test even beyond the linked years in this study, the follow-up is insufficient to assess long-term prognostic value with survival outcomes. Fourth, other key endpoints, including biochemical failure, local recurrence, and distant metastasis, are not available in either of the linked registries. Finally, SEER does not contain information on use of specific imaging modalities (eg, magnetic resonance imaging), which could impact treatment decisions (19,20).

In the United States, there was a correlation between GC scores and NCCN risk groups. There is an association between the use of the biopsy GC test and likelihood to undergo conservative management. Additionally, higher genomic classifier scores are associated with higher rates of adverse pathology at the time of surgery and greater use of postoperative radiotherapy.

## Supplementary Material

pkad052_Supplementary_Data

## Data Availability

Data from linkage of SEER cancer registries cases to Decipher Prostate test results will be made available to the research community as a SEER Specialized Database at -https://seer.cancer.gov/data-software/specialized.html. Please contact valentina.petkov@nih.gov with any questions about data access.

## References

[1] Dess RT , Suresh K, Zelefsky MJ, et al Development and validation of a clinical prognostic stage group system for nonmetastatic prostate cancer using disease-specific mortality results from the International Staging Collaboration for Cancer of the Prostate. JAMA Oncol. 2020;6(12):1912-1920. doi: 10.1001/jamaoncol.2020.4922. 33090219 PMC7582232

[2] Jairath NK , Dal Pra A, Vince R Jr, et al A systematic review of the evidence for the decipher genomic classifier in prostate cancer. Eur Urol. 2021;79(3):374-383. 33293078 10.1016/j.eururo.2020.11.021

[3] Feng FY , Thomas S, Saad F, et al Association of molecular subtypes with differential outcome to apalutamide treatment in nonmetastatic castration-resistant prostate cancer. JAMA Oncol. 2021;7(7):1005-1014. doi:10.1001/jamaoncol.2021.1463. 34081076 PMC8176389

[4] Feng FY , Huang HC, Spratt DE, et al Validation of a 22-gene genomic classifier in patients with recurrent prostate cancer: an ancillary study of the NRG/RTOG 9601 randomized clinical trial. JAMA Oncol 2021;7(4):544-552. doi:10.1001/jamaoncol.2020.7671. Erratum in: *JAMA Oncol.* 2021;7(4):639. 33570548 PMC7879385

[5] Attard G , Parry M, Grist E, et al Clinical testing of transcriptome-wide expression profiles in high-risk localized and metastatic prostate cancer starting androgen deprivation therapy: an ancillary study of the STAMPEDE abiraterone Phase 3 trial. Preprint. *Res Sq*. 2023;rs.3.rs-2488586. Published 2023 Feb 8. doi:10.21203/rs.3.rs-2488586/v1.

[6] Hamid AA , Huang HC, Wang V, et al Transcriptional profiling of primary prostate tumor in metastatic hormone-sensitive prostate cancer and association with clinical outcomes: correlative analysis of the E3805 CHAARTED trial. Ann Oncol 2021;32(9):1157-1166. doi:10.1016/j.annonc.2021.06.003. 34129855 PMC8463957

[7] Cooperberg MR , Carroll PR, Dall'Era MA, et al The state of the science on prostate cancer biomarkers: the San Francisco consensus statement. Eur Urol. 2019;76(3):268-272. 31128968 10.1016/j.eururo.2019.05.013

[8] Simon RM , Paik S, Hayes DF. Use of archived specimens in evaluation of prognostic and predictive biomarkers. J Natl Cancer Inst. 2009;101(21):1446-1452. 19815849 10.1093/jnci/djp335 PMC2782246

[9] Gore JL , Du Plessis M, Zhang J, et al Clinical utility of a genomic classifier in men undergoing radical prostatectomy: the PRO-IMPACT trial. Pract Radiat Oncol. 2020;10(2):e82-e90. 31761540 10.1016/j.prro.2019.09.016

[10] Howlader N , Noone AM, Krapcho M, et al, eds. *SEER Cancer Statistics Review, 1975-2018*. Bethesda, MD: National Cancer Institute. https://seer.cancer.gov/csr/1975_2018/, based on November 2020 SEER data submission, posted to the SEER web site, April 2021.

[11] Duggan MA , Anderson WF, Altekruse S, Penberthy L, Sherman ME. The Surveillance, Epidemiology, and End Results (SEER) program and pathology: toward strengthening the critical relationship. Am J Surg Pathol. 2016;40(12):e94-e102. doi:10.1097/PAS.0000000000000749. PMID: 27740970; PMCID: PMC5106320. 27740970 PMC5106320

[12] Schaeffer EM , Srinivas S, Antonarakis ES, et al NCCN guidelines insights: prostate cancer, version 1.2021. J Natl Compr Canc Netw. 2021;19(2):134-143. doi: 10.6004/jnccn.2021.0008. PMID: 33545689. 33545689

[13] Ross AE , Johnson MH, Yousefi K, et al Tissue-based genomics augments post-prostatectomy risk stratification in a natural history cohort of intermediate- and high-risk men. Eur Urol. 2016;69(1):157-165. 26058959 10.1016/j.eururo.2015.05.042

[14] Yost K , Perkins C, Cohen R, Morris C, Wright W. Socioeconomic status and breast cancer incidence in California for different race/ethnic groups. Cancer Causes Control. 2001;12(8):703-711. 11562110 10.1023/a:1011240019516

[15] Brooks MA , Thomas L, Magi-Galluzzi C, et al Validating the association of adverse pathology with distant metastasis and prostate cancer mortality 20-years after radical prostatectomy. Urol Oncol 2022;40(3):104.e1-104.e7. doi:10.1016/j.urolonc.2021.10.005. 34824014

[16] Hu JC , Tosoian JJ, Qi J, et al Clinical utility of gene expression classifiers in men with newly diagnosed prostate cancer. JCO Precis Oncol. 2018;2:PO.18.00163. doi:10.1200/po.18.00163 PMC7440129 32832833

[17] Herlemann A , Huang HC, Alam R, et al Decipher identifies men with otherwise clinically favorable-intermediate risk disease who may not be good candiddates for active surveillance. Prostate Cancer Prostatic Dis. 2020;23(1):136-143. 31455846 10.1038/s41391-019-0167-9 PMC8076042

[18] Spratt DE , Chan T, Waldron L, et al Racial/ethnic disparities in genomic sequencing. JAMA Oncol. 2016;2(8):1070-1074. 27366979 10.1001/jamaoncol.2016.1854 PMC5123755

[19] Jambor I , Falagario U, Ratnani P, et al Prediction of biochemical recurrence in prostate cancer patients who underwent prostatectomy using routine clinical prostate multiparametric MRI and decipher genomic score. J Magn Reson Imaging 2020;51(4):1075-1085. 31566845 10.1002/jmri.26928

[20] Vince RA Jr , Jiang R, Qi J, et al Impact of Decipher Biopsy testing on clinical outcomes in localized prostate cancer in a prospective statewide collaborative. Prostate Cancer Prostatic Dis. 2021;25(4):677-683. doi:10.1038/s41391-021-00428-y. 34285350 PMC8770695

