# Supplementary material for: Use of the Decipher genomic classifier among men with prostate cancer in the United States
Source: JNCI Cancer Spectr. 2023 Aug 1;7(5):pkad052. doi: 10.1093/jncics/pkad052 (PMC10505256; doi:10.1093/jncics/pkad052)

Decipher-SEER Linkage Supplementary Materials

Supplementary Tables/Figures

- Supplementary Table 1: Demographics of GC tested and untested patient cohorts.
- Supplementary Table 2: Clinical and pathological characteristics of GC tested patients stratified by test type and GC risk group.
- Supplementary Table 3: Treatment information in the GC biopsy tested cohort.
- Supplementary Table 4A/B: Multivariable (MVA) logistic regression for AS/WW between GC biopsy tested and untested cohorts (both restricted to the time frame the GC biopsy test was available (2016 onward) and among all patients).
- Supplementary Table 5: Multivariable logistic regression for a more stringent definition of adverse pathology (pN+, pT3/4, or pGG4-5) in NCCN very low/low/favorable-intermediate risk men tested with GC who were subsequently treated with prostatectomy.
- Supplementary Table 6: Treatment information in the GC post-op tested cohort.
- Supplementary Table 7: Multivariable logistic regression results for use of radiation therapy after radical prostatectomy in men with GC prostatectomy and adverse pathologic features (pN+, pT3/4, or pGG4-5).
- Supplementary Figure 1: Patient demographic information in test ordered and unordered cohorts.
- Supplementary Figure 2A/B: Boxplots of (A) GC biopsy scores and (B) GC prostatectomy scores by clinicopathologic features.

Supplementary Table 1: Demographics of GC tested and untested patient cohorts.

Variables	GC Tested		Untested
	Biopsy	Post-Op	
**N**	3949 (0.7)	4978 (0.9)	563618 (98.4)
**Age**			
Median (IQR)			
40-44 years	7 (0.2)	24 (0.5)	2213 (0.4)
45-49 years	62 (1.6)	131 (2.6)	10447 (1.9)
50-54 years	247 (6.3)	427 (8.6)	36731 (6.5)
55-59 years	542 (13.7)	794 (16.0)	74148 (13.2)
60-64 years	793 (20.1)	1153 (23.2)	107906 (19.1)
65-69 years	1005 (25.4)	1472 (29.6)	127680 (22.7)
70-74 years	753 (19.1)	765 (15.4)	93688 (16.6)
75-79 years	407 (10.3)	187 (3.8)	57747 (10.2)
80-84 years	111 (2.8)	22 (0.4)	30087 (5.3)
85+ years	22 (0.6)	3 (0.1)	22971 (4.1)
**Race / Ethnicity**			
Non-Hispanic White	2922 (74.0)	3836 (77.1)	379795 (67.4)
Non-Hispanic Black	425 (10.8)	518 (10.4)	84894 (15.1)
Hispanic (All Races)	270 (6.8)	372 (7.5)	56196 (10.0)
Non-Hispanic Asian or Pacific Islander	152 (3.8)	185 (3.7)	25985 (4.6)
Non-Hispanic Unknown Race	174 (4.4)	57 (1.1)	14920 (2.6)
Non-Hispanic American Indian/Alaska Native	6 (0.2)	10 (0.2)	1828 (0.3)
**Marital Status**			
Married	2625 (66.5)	3816 (76.7)	347892 (61.7)
Separated	325 (8.2)	395 (7.9)	68392 (12.1)
Single	372 (9.4)	517 (10.4)	66297 (11.8)
Unknown	627 (15.9)	250 (5.0)	81037 (14.4)
**Census Tract SES Quintile**			
1	261 (6.6)	392 (7.9)	74353 (13.2)
2	392 (9.9)	546 (11.0)	86396 (15.3)
3	543 (13.8)	746 (15.0)	100834 (17.9)
4	924 (23.4)	1118 (22.5)	126968 (22.5)
5	1714 (43.4)	2000 (40.2)	161362 (28.6)
Unavailable	115 (2.9)	176 (3.5)	13705 (2.4)
**Diagnosis Year**			
2010	1 (0.0)	22 (0.4)	69324 (12.3)
2011	0 (0.0)	88 (1.8)	70310 (12.5)
2012	8 (0.2)	137 (2.8)	60564 (10.7)
2013	12 (0.3)	229 (4.6)	58971 (10.5)
2014	23 (0.6)	326 (6.5)	55523 (9.9)
2015	44 (1.1)	761 (15.3)	58539 (10.4)
2016	713 (18.1)	1334 (26.8)	60224 (10.7)
2017	1874 (47.5)	1213 (24.4)	64531 (11.4)
2018	1274 (32.3)	868 (17.4)	65632 (11.6)
**GC Test Year**			
2014	0 (0.0)	4 (0.1)	
2015	0 (0.0)	16 (0.3)	
2016	328 (8.3)	1591 (32.0)	
2017	2016 (51.1)	1654 (33.2)	
2018	1303 (33.0)	1097 (22.0)	
2019	272 (6.9)	494 (9.9)	
2020	30 (0.8)	122 (2.5)	
**GC Score**			
Median (Q1, Q3)	0.5 (0.34, 0.65)	0.58 (0.4, 0.75)	
Low	1626 (41.2)	1473 (29.6)	
Intermediate	1084 (27.4)	1175 (23.6)	
High	1239 (31.4)	2330 (46.8)	

Supplemental Table 2: Clinical and Pathological characteristics of patients stratified by test type.

	GC Biopsy Risk Group					GC RP Risk Group				
	Low	Intermediate	High	Overall	p	Low	Intermediate	High	Overall	p
**N (%)**	1626 (41.2)	1084 (27.4)	1239 (31.4)	3949		1473 (29.6)	1175 (23.6)	2330 (46.8)	4978	
**Clinical Grade Group**										
1	788 (48.5)	429 (39.6)	266 (21.5)	1483 (37.6)	<0.001[a]	355 (24.1)	198 (16.9)	151 (6.5)	704 (14.1)	<0.001[a]
2	580 (35.7)	430 (39.7)	454 (36.6)	1464 (37.1)		562 (38.2)	397 (33.8)	591 (25.4)	1550 (31.1)	
3	136 (8.4)	122 (11.3)	256 (20.7)	514 (13.0)		259 (17.6)	255 (21.7)	557 (23.9)	1071 (21.5)	
4	35 (2.2)	44 (4.1)	107 (8.6)	186 (4.7)		149 (10.1)	168 (14.3)	480 (20.6)	797 (16.0)	
5	11 (0.7)	25 (2.3)	124 (10.0)	160 (4.1)		54 (3.7)	100 (8.5)	437 (18.8)	591 (11.9)	
Unavailable	76 (4.7)	34 (3.1)	32 (2.6)	142 (3.6)		94 (6.4)	57 (4.9)	114 (4.9)	265 (5.3)	
**Clinical Stage**										
T1	1047 (64.4)	699 (64.5)	688 (55.5)	2434 (61.6)	<0.001[a]	972 (66.0)	704 (59.9)	1199 (51.5)	2875 (57.8)	<0.001[a]
T2	380 (23.4)	268 (24.7)	385 (31.1)	1033 (26.2)		376 (25.5)	331 (28.2)	723 (31.0)	1430 (28.7)	
T3a	20 (1.2)	16 (1.5)	29 (2.3)	65 (1.6)		31 (2.1)	56 (4.8)	185 (7.9)	272 (5.5)	
T3b	2 (0.1)	4 (0.4)	32 (2.6)	38 (1.0)		14 (1.0)	23 (2.0)	107 (4.6)	144 (2.9)	
T3NOS	1 (0.1)	3 (0.3)	10 (0.8)	14 (0.4)		10 (0.7)	15 (1.3)	25 (1.1)	50 (1.0)	
T4	0 (0.0)	0 (0.0)	13 (1.0)	13 (0.3)		1 (0.1)	1 (0.1)	10 (0.4)	12 (0.2)	
Unavailable	176 (10.8)	94 (8.7)	82 (6.6)	352 (8.9)		69 (4.7)	45 (3.8)	81 (3.5)	195 (3.9)	
**Percent Positive Cores**										
Median (Q1, Q3)	0.25 (0.17, 0.44)	0.33 (0.17, 0.43)	0.36 (0.2, 0.58)	0.33 (0.17, 0.5)	<0.001[b]	0.42 (0.25, 0.58)	0.42 (0.25, 0.593)	0.50 (0.33, 0.69)	0.43 (0.25, 0.67)	<0.001[b]
**Prostate Specific Antigen**										
Median (Q1, Q3)	6.1 (4.8, 8.1)	6.3 (5, 8.75)	7.0 (5.2, 10.6)	6.4 (5, 9)	<0.001[b]	6.1 (4.8, 8.8)	6.9 (5.2, 10.5)	7.4 (5.4, 11.6)	6.9 (5.1, 10.5)	<0.001[b]
PSA < 10	1170 (72.0)	759 (70.0)	809 (65.3)	2738 (69.3)	<0.001[a]	1051 (71.4)	765 (65.1)	1460 (62.7)	3276 (65.8)	<0.001[a]
10 ≤ PSA ≤ 20	168 (10.3)	127 (11.7)	218 (17.6)	513 (13.0)		192 (13.0)	228 (19.4)	470 (20.2)	890 (17.9)	
PSA > 20	42 (2.6)	53 (4.9)	89 (7.2)	184 (4.7)		64 (4.3)	66 (5.6)	221 (9.5)	351 (7.1)	
Unavailable	246 (15.1)	145 (13.4)	123 (9.9)	514 (13.0)		166 (11.3)	116 (9.9)	179 (7.7)	461 (9.3)	
**NCCN Risk Group**										
Very Low / Low Risk	546 (33.6)	287 (26.5)	173 (14.0)	1006 (25.5)	<0.001[a]	248 (16.8)	114 (9.7)	97 (4.2)	459 (9.2)	<0.001[a]
Favorable-Intermediate Risk	323 (19.9)	227 (20.9)	219 (17.7)	769 (19.5)		200 (13.6)	136 (11.6)	160 (6.9)	496 (10.0)	
Intermediate (NOS) Risk	66 (4.1)	53 (4.9)	39 (3.1)	158 (4.0)		84 (5.7)	56 (4.8)	81 (3.5)	221 (4.4)	
Unfavorable-Intermediate Risk	318 (19.6)	241 (22.2)	393 (31.7)	952 (24.1)		458 (31.1)	375 (31.9)	647 (27.8)	1480 (29.7)	
High / Very High Risk	103 (6.3)	118 (10.9)	244 (19.7)	465 (11.8)		282 (19.1)	376 (32.0)	1124 (48.2)	1782 (35.8)	
Unavailable	270 (16.6)	158 (14.6)	171 (13.8)	599 (15.2)		201 (13.6)	118 (10.0)	221 (9.5)	540 (10.8)	
**Pathological Grade Group**										
1	39 (2.4)	26 (2.4)	27 (2.2)	92 (2.3)	<0.001[a]	135 (9.2)	38 (3.2)	27 (1.2)	200 (4.0)	<0.001[a]
2	224 (13.8)	186 (17.2)	231 (18.6)	641 (16.2)		847 (57.5)	548 (46.6)	643 (27.6)	2038 (40.9)	
3	48 (3.0)	68 (6.3)	120 (9.7)	236 (6.0)		277 (18.8)	336 (28.6)	782 (33.6)	1395 (28.0)	
4	8 (0.5)	7 (0.6)	18 (1.5)	33 (0.8)		70 (4.8)	69 (5.9)	234 (10.0)	373 (7.5)	
5	3 (0.2)	10 (0.9)	47 (3.8)	60 (1.5)		49 (3.3)	118 (10.0)	531 (22.8)	698 (14.0)	
Unavailable	1304 (80.2)	787 (72.6)	796 (64.2)	2887 (73.1)		95 (6.4)	66 (5.6)	113 (4.8)	274 (5.5)	
**Pathological Stage**										
T2	248 (15.3)	219 (20.2)	260 (21.0)	727 (18.4)	<0.001[a]	794 (53.9)	453 (38.6)	572 (24.5)	1819 (36.5)	<0.001[a]
T3a	62 (3.8)	58 (5.4)	112 (9.0)	232 (5.9)		444 (30.1)	448 (38.1)	871 (37.4)	1763 (35.4)	
T3b	11 (0.7)	21 (1.9)	62 (5.0)	94 (2.4)		114 (7.7)	173 (14.7)	646 (27.7)	933 (18.7)	
T3NOS	2 (0.1)	4 (0.4)	7 (0.6)	13 (0.3)		13 (0.9)	19 (1.6)	78 (3.3)	110 (2.2)	
T4	4 (0.2)	1 (0.1)	3 (0.2)	8 (0.2)		1 (0.1)	6 (0.5)	34 (1.5)	41 (0.8)	
Unavailable	1299 (79.9)	781 (72.0)	795 (64.2)	2875 (72.8)		107 (7.3)	76 (6.5)	129 (5.5)	312 (6.3)	
**Lymph Node Invasion**										
No/Unknown	1624 (99.9)	1080 (99.6)	1188 (95.9)	3892 (98.6)	<0.001[a]	1452 (98.6)	1143 (97.3)	2166 (93.0)	4761 (95.6)	<0.001
Yes	2 (0.1)	4 (0.4)	51 (4.1)	57 (1.4)		21 (1.4)	32 (2.7)	164 (7.0)	217 (4.4)	
**De novo metastatic**										
No	1535 (94.4)	1012 (93.4)	1097 (88.5)	3644 (92.3)	<0.001[a]	1397 (94.8)	1119 (95.2)	2210 (94.8)	4726 (94.9)	<0.001[a]
Yes	1 (0.1)	5 (0.5)	36 (2.9)	42 (1.1)		3 (0.2)	3 (0.3)	32 (1.4)	38 (0.8)	
Unavailable	90 (5.5)	67 (6.2)	106 (8.6)	263 (6.7)		73 (5.0)	53 (4.5)	88 (3.8)	214 (4.3)	

[a]Chi-squared test
[b]Kruskal-Wallis test
GC, genomic classifier; NOS, not otherwise specified; PSA, prostate specific antigen; NCCN, National Comprehensive Cancer Network; RP, Radical Prostatectomy

Supplementary Table 3: Treatment information in the GC biopsy tested cohort.

Variables	Decipher Biopsy Risk Group: Low	Intermediate	High	Overall	P-value
**Total**	1626 (41.2)	1084 (27.4)	1239 (31.4)	3949 (100.0)	
**Active Surveillance / Watchful Waiting**					
No/Unknown	1036 (63.7)	813 (75.0)	1114 (89.9)	2963 (75.0)	<0.001[a]
Yes	590 (36.3)	271 (25.0)	125 (10.1)	986 (25.0)	
**Cancer-directed Surgery**					
Not recommended	1142 (70.2)	686 (63.3)	690 (55.7)	2518 (63.8)	<0.001[a]
Surgery performed	358 (22.0)	326 (30.1)	484 (39.1)	1168 (29.6)	
Recommended but not performed	32 (2.0)	20 (1.8)	23 (1.9)	75 (1.9)	
Unavailable	94 (5.8)	52 (4.8)	42 (3.4)	188 (4.8)	
**Radiation Therapy**					
No/Unknown	1324 (81.4)	800 (73.8)	773 (62.4)	2897 (73.4)	<0.001[a]
Yes	264 (16.2)	265 (24.4)	427 (34.5)	956 (24.2)	
Unavailable	38 (2.3)	19 (1.8)	39 (3.1)	96 (2.4)	
**Radiation Recode**					
None/Unknown	1324 (81.4)	800 (73.8)	773 (62.4)	2897 (73.4)	<0.001[a]
Beam radiation	175 (10.8)	173 (16.0)	328 (26.5)	676 (17.1)	
Radioactive implants (includes brachytherapy) (1988+)	63 (3.9)	53 (4.9)	51 (4.1)	167 (4.2)	
Combination of beam with implants or isotopes	23 (1.4)	37 (3.4)	48 (3.9)	108 (2.7)	
Radiation, NOS method or source not specified	1 (0.1)	2 (0.2)	0 (0.0)	3 (0.1)	
Radioisotopes (1988+)	2 (0.1)	0 (0.0)	0 (0.0)	2 (0.1)	
Refused (1988+)	22 (1.4)	6 (0.6)	15 (1.2)	43 (1.1)	
Recommended, unknown if administered	16 (1.0)	13 (1.2)	24 (1.9)	53 (1.3)	
**Chemotherapy**					
No/Unknown	1626 (100.0)	1081 (99.7)	1222 (98.6)	3929 (99.5)	<0.001[a]
Yes	0 (0.0)	3 (0.3)	17 (1.4)	20 (0.5)	
**Surgery / Radiation Seq.**					
No radiation and/or cancer-directed surgery	1621 (99.7)	1071 (98.8)	1196 (96.5)	3888 (98.5)	<0.001[a]
Radiation after surgery	5 (0.3)	12 (1.1)	40 (3.2)	57 (1.4)	
Radiation prior to surgery	0 (0.0)	1 (0.1)	2 (0.2)	3 (0.1)	
Sequence unknown, but both were given	0 (0.0)	0 (0.0)	1 (0.1)	1 (0.0)	
**Systemic / Surgery Seq.**					
No systemic therapy and/or surgical procedures	1617 (99.4)	1071 (98.8)	1189 (96.0)	3877 (98.2)	<0.001[a]
Systemic therapy after surgery	5 (0.3)	5 (0.5)	30 (2.4)	40 (1.0)	
Systemic therapy both before and after surgery	1 (0.1)	0 (0.0)	9 (0.7)	10 (0.3)	
Systemic therapy before surgery	3 (0.2)	7 (0.6)	10 (0.8)	20 (0.5)	
Surgery both before and after systemic therapy	0 (0.0)	0 (0.0)	1 (0.1)	1 (0.0)	
Sequence unknown	0 (0.0)	1 (0.1)	0 (0.0)	1 (0.0)	

[a]Chi-squared test

Supplementary Table 4A: Multivariable (MVA) logistic regression for AS/WW between GC biopsy tested and untested populations.

Model	Variable	Odds Ratio (95% CI)	P-value	# of patients	# of events
MVA: Test Ordering + Age (per 5 years) + Diagnosis Year + Race + Marital Status + Yost US Quintile + Log2(PSA) + PPC (per 0.1) + Clinical GG + Clinical Stage	GC Biopsy Ordered	2.21 (2.05 - 2.39)	<0.001*	284398	35994
	Age (per 5 years)	1.16 (1.15 - 1.17)	<0.001*		
	Diagnosis Year: 2011 vs. 2010	1.33 (1.25 - 1.41)	<0.001*		
	Diagnosis Year: 2012 vs. 2010	1.81 (1.70 - 1.93)	<0.001*		
	Diagnosis Year: 2013 vs. 2010	2.39 (2.25 - 2.54)	<0.001*		
	Diagnosis Year: 2014 vs. 2010	2.90 (2.72 - 3.08)	<0.001*		
	Diagnosis Year: 2015 vs. 2010	3.68 (3.46 - 3.91)	<0.001*		
	Diagnosis Year: 2016 vs. 2010	4.37 (4.12 - 4.64)	<0.001*		
	Diagnosis Year: 2017 vs. 2010	4.67 (4.41 - 4.95)	<0.001*		
	Diagnosis Year: 2018 vs. 2010	5.35 (5.05 - 5.68)	<0.001*		
	Race: NH Black vs. NH White	0.93 (0.89 - 0.97)	<0.001*		
	Race: NH Asian / Pacific Islander vs. NH White	1.03 (0.97 - 1.10)	0.33		
	Race: Hispanic (All Races) vs. NH White	0.90 (0.86 - 0.94)	<0.001*		
	Race: NH Other / Unknown vs. NH White	0.95 (0.87 - 1.04)	0.30		
	Marital Status: Separated (vs. Married)	1.36 (1.31 - 1.42)	<0.001*		
	Marital Status: Single (vs. Married)	1.25 (1.20 - 1.30)	<0.001*		
	Marital Status: Unknown (vs. Married)	1.23 (1.18 - 1.29)	<0.001*		
	Yost US Index Quintile 2 (vs. 1)	1.20 (1.14 - 1.26)	<0.001*		
	Yost US Index Quintile 3 (vs. 1)	1.29 (1.23 - 1.36)	<0.001*		
	Yost US Index Quintile 4 (vs. 1)	1.41 (1.34 - 1.48)	<0.001*		
	Yost US Index Quintile 5 (vs. 1)	1.63 (1.55 - 1.71)	<0.001*		
	Log2(PSA)	0.91 (0.90 - 0.92)	<0.001*		
	PPC (per 0.1)	0.75 (0.75 - 0.76)	<0.001*		
	Cling. GG 2 (vs. 1)	0.17 (0.16 - 0.17)	<0.001*		
	Cling. GG 3 (vs. 1)	0.08 (0.07 - 0.08)	<0.001*		
	Cling. GG 4 (vs. 1)	0.03 (0.03 - 0.03)	<0.001*		
	Cling. GG 5 (vs. 1)	0.02 (0.02 - 0.03)	<0.001*		
	Clinical Stage T2 (vs. T1)	0.74 (0.72 - 0.76)	<0.001*		
	Clinical Stage T3/4 (vs. T1)	0.13 (0.10 - 0.17)	<0.001*		

* indicates p-value < 0.05.

Supplementary Table 4B: Multivariable (MVA) logistic regression for AS/WW between GC biopsy tested and untested populations (restricted to patients with year of diagnosis 2016 onward).

Model	Variable	Odds Ratio (95% CI)	P-value	# of patients	# of events
MVA: Test Ordering + Age (per 5 years) + Diagnosis Year + Race + Marital Status + Yost US Quintile + Log2(PSA) + PPC (per 0.1) + Clinical GG + Clinical Stage	GC Biopsy Ordered	2.05 (1.88 - 2.23)	<0.001*	102924	16633
	Age (per 5 years)	1.13 (1.12 - 1.15)	<0.001*		
	Diagnosis Year: 2017 vs. 2016	1.08 (1.02 - 1.13)	0.004*		
	Diagnosis Year: 2018 vs. 2016	1.26 (1.20 - 1.33)	<0.001*		
	Race: NH Black vs. NH White	0.86 (0.81 - 0.91)	<0.001*		
	Race: NH Asian / Pacific Islander vs. NH White	0.98 (0.89 - 1.09)	0.74		
	Race: Hispanic (All Races) vs. NH White	0.86 (0.80 - 0.93)	<0.001*		
	Race: NH Other / Unknown vs. NH White	0.72 (0.62 - 0.83)	<0.001*		
	Marital Status: Separated (vs. Married)	1.25 (1.17 - 1.34)	<0.001*		
	Marital Status: Single (vs. Married)	1.23 (1.16 - 1.31)	<0.001*		
	Marital Status: Unknown (vs. Married)	0.91 (0.85 - 0.98)	0.01*		
	Yost US Index Quintile 2 (vs. 1)	1.24 (1.15 - 1.35)	<0.001*		
	Yost US Index Quintile 3 (vs. 1)	1.32 (1.22 - 1.43)	<0.001*		
	Yost US Index Quintile 4 (vs. 1)	1.42 (1.32 - 1.53)	<0.001*		
	Yost US Index Quintile 5 (vs. 1)	1.65 (1.54 - 1.78)	<0.001*		
	Log2(PSA)	0.89 (0.87 - 0.91)	<0.001*		
	PPC (per 0.1)	0.75 (0.74 - 0.76)	<0.001*		
	Cling. GG 2 (vs. 1)	0.13 (0.13 - 0.14)	<0.001*		
	Cling. GG 3 (vs. 1)	0.05 (0.05 - 0.06)	<0.001*		
	Cling. GG 4 (vs. 1)	0.02 (0.02 - 0.02)	<0.001*		
	Cling. GG 5 (vs. 1)	0.02 (0.01 - 0.02)	<0.001*		
	Clinical Stage T2 (vs. T1)	0.62 (0.59 - 0.66)	<0.001*		
	Clinical Stage T3/4 (vs. T1)	0.12 (0.08 - 0.17)	<0.001*		

* indicates p-value < 0.05.

Supplementary Table 5: Multivariable logistic regression results for a more stringent definition of adverse pathology (pN+, pT3/4, or pGG4-5) in NCCN Very Low / Low / Favorable-Intermediate risk men with GC biopsy testing who were subsequently treated with RP.

Model	Variable	Odds Ratio (95% CI)	P-value	Odds Ratio (95% CI)	P-value	# of patients	# of events
MVA: GC + PSA + PPC (per 0.1) + Clinical GG + Clinical Stage	GC Score (Continuous)	1.29 (1.09 - 1.52)	0.009*[a]	-	-	361	55
	GC Risk Group: Int. vs. Low	-	-	1.56 (0.67 - 3.64)	0.30[a]		
	GC Risk Group: High vs. Low	-	-	2.88 (1.35 - 6.17)	0.01*[a]		
	Age (per 5 years)	1.16 (0.92 - 1.46)	0.20	1.18 (0.93 - 1.48)	0.17		
	Log2(PSA)	1.35 (0.79 - 2.34)	0.28	1.35 (0.78 - 2.33)	0.29		
	PPC (per 0.1)	1.01 (0.82 - 1.24)	0.93	1.01 (0.83 - 1.24)	0.90		
	Cling. GG 2 (vs. 1)	0.94 (0.51 - 1.73)	0.84	0.96 (0.52 - 1.77)	0.90		
	Clinical Stage T2 (vs. T1)	1.42 (0.71 - 2.86)	0.32	1.38 (0.68 - 2.77)	0.37		

Odds ratios of genomic classifiers are per 0.1 unit increase. * indicates p-value < 0.05. [a]Bonferroni-Holm adjusted p-values.

Supplementary Table 6: Treatment information in the GC post-op tested cohort.

Variables	Decipher Post-Op Risk Group			Overall	P-value
	Low	Intermediate	High		
**Total**	1473 (29.6)	1175 (23.6)	2330 (46.8)	4978 (100.0)	
**Active Surveillance / Watchful Waiting**					
No/Unknown	1451 (98.5)	1153 (98.1)	2302 (98.8)	4906 (98.6)	0.287[a]
Yes	22 (1.5)	22 (1.9)	28 (1.2)	72 (1.4)	
**Cancer-directed Surgery**					
Not recommended	50 (3.4)	33 (2.8)	50 (2.1)	133 (2.7)	0.152[a]
Surgery performed	1410 (95.7)	1129 (96.1)	2258 (96.9)	4797 (96.4)	
Recommended but not performed	6 (0.4)	2 (0.2)	8 (0.3)	16 (0.3)	
Unavailable	7 (0.5)	11 (0.9)	14 (0.6)	32 (0.6)	
**Radiation Therapy**					
No/Unknown	1372 (93.1)	1034 (88.0)	1760 (75.5)	4166 (83.7)	<0.001[a]
Yes	60 (4.1)	96 (8.2)	476 (20.4)	632 (12.7)	
Unavailable	41 (2.8)	45 (3.8)	94 (4.0)	180 (3.6)	
**Radiation Recode**					
None/Unknown	1372 (93.1)	1034 (88.0)	1760 (75.5)	4166 (83.7)	<0.001[a]
Beam radiation	59 (4.0)	95 (8.1)	471 (20.2)	625 (12.6)	
Combination of beam with implants or isotopes	0 (0.0)	0 (0.0)	1 (0.0)	1 (0.0)	
Radiation, NOS method or source not specified	1 (0.1)	1 (0.1)	4 (0.2)	6 (0.1)	
Refused (1988+)	18 (1.2)	12 (1.0)	31 (1.3)	61 (1.2)	
Recommended, unknown if administered	23 (1.6)	33 (2.8)	63 (2.7)	119 (2.4)	
**Chemotherapy**					
No/Unknown	1471 (99.9)	1171 (99.7)	2311 (99.2)	4953 (99.5)	0.010[a]
Yes	2 (0.1)	4 (0.3)	19 (0.8)	25 (0.5)	
**Surgery / Radiation Seq.**					
No radiation and/or cancer-directed surgery	1413 (95.9)	1077 (91.7)	1854 (79.6)	4344 (87.3)	<0.001[a]
Radiation after surgery	59 (4.0)	95 (8.1)	470 (20.2)	624 (12.5)	
Radiation prior to surgery	0 (0.0)	1 (0.1)	4 (0.2)	5 (0.1)	
Radiation before and after surgery	0 (0.0)	0 (0.0)	1 (0.0)	1 (0.0)	
Sequence unknown, but both were given	1 (0.1)	2 (0.2)	1 (0.0)	4 (0.1)	
**Systemic / Surgery Seq.**					
No systemic therapy and/or surgical procedures	1440 (97.8)	1101 (93.7)	1857 (79.7)	4398 (88.3)	<0.001[a]
Systemic therapy after surgery	21 (1.4)	51 (4.3)	368 (15.8)	440 (8.8)	
Systemic therapy both before and after surgery	1 (0.1)	7 (0.6)	42 (1.8)	50 (1.0)	
Systemic therapy before surgery	7 (0.5)	13 (1.1)	53 (2.3)	73 (1.5)	
Sequence unknown	4 (0.3)	3 (0.3)	9 (0.4)	16 (0.3)	
Intraoperative systemic therapy	0 (0.0)	0 (0.0)	1 (0.0)	1 (0.0)	

[a]Chi-squared test

Supplementary Table 7: Multivariable logistic regression results for use of radiation therapy after radical prostatectomy in GC RP tested men and adverse pathologic features (pN+, pT3/4, or pGG4-5).

		Continuous GC MVA		Categorical GC MVA			
Model	Variable	Odds Ratio (95% CI)	P-value	Odds Ratio (95% CI)	P-value	# of patients	# of events
MVA: GC Score (Continuous) + Age + Diagnosis Year + Race + Marital Status + Census Tract SES Quintile + PSA + Pathological Grade + Pathological Stage + LNI	GC Score (Continuous)	1.20 (1.13 - 1.28)	<0.001*[a]	-	-	2745	504
	GC Risk Group: Int. vs. Low	-	-	1.58 (1.05 - 2.35)	0.03*[a]		
	GC Risk Group: High vs. Low	-	-	2.72 (1.91 - 3.87)	<0.001*[a]		
	Age (per 5 years)	0.89 (0.82 - 0.96)	0.002*	0.88 (0.82 - 0.95)	0.001*		
	Diagnosis Year: 2016 vs. ≤ 2015	1.10 (0.81 - 1.48)	0.55	1.08 (0.80 - 1.46)	0.62		
	Diagnosis Year: 2017 vs. ≤ 2015	1.10 (0.82 - 1.49)	0.52	1.12 (0.83 - 1.51)	0.47		
	Diagnosis Year: 2018 vs. ≤ 2015	1.26 (0.92 - 1.73)	0.16	1.29 (0.94 - 1.76)	0.12		
	Race: NH Black vs. NH White	0.95 (0.65 - 1.40)	0.81	0.95 (0.65 - 1.41)	0.81		
	Race: NH Asian / Pacific Islander vs. NH White	1.46 (0.91 - 2.35)	0.12	1.41 (0.87 - 2.26)	0.16		
	Race: Hispanic (All Races) vs. NH White	1.06 (0.71 - 1.58)	0.77	1.08 (0.73 - 1.61)	0.69		
	Race: NH Other / Unknown vs. NH White	0.86 (0.25 - 3.00)	0.81	0.86 (0.25 - 3.01)	0.82		
	Marital Status: Separated (vs. Married)	0.63 (0.41 - 0.95)	0.03*	0.64 (0.42 - 0.98)	0.04*		
	Marital Status: Single (vs. Married)	1.23 (0.89 - 1.70)	0.22	1.21 (0.88 - 1.68)	0.24		
	Marital Status: Unknown (vs. Married)	0.61 (0.33 - 1.16)	0.13	0.61 (0.32 - 1.15)	0.12		
	Census Tract SES Quintile 2 (vs. 1)	0.76 (0.47 - 1.23)	0.27	0.77 (0.48 - 1.25)	0.29		
	Census Tract SES Quintile 3 (vs. 1)	0.70 (0.44 - 1.12)	0.14	0.69 (0.43 - 1.10)	0.12		
	Census Tract SES Quintile 4 (vs. 1)	0.70 (0.45 - 1.09)	0.11	0.71 (0.46 - 1.10)	0.12		
	Census Tract SES Quintile 5 (vs. 1)	0.68 (0.45 - 1.04)	0.07	0.68 (0.45 - 1.03)	0.07		
	Log2(PSA)	1.18 (1.07 - 1.31)	<0.001*	1.18 (1.07 - 1.30)	0.001*		
	Pathological Grade 2 vs. 1	1.32 (0.39 - 4.42)	0.65	1.23 (0.36 - 4.12)	0.74		
	Pathological Grade 3 vs. 1	2.02 (0.61 - 6.75)	0.25	1.93 (0.58 - 6.47)	0.29		
	Pathological Grade 4 vs. 1	2.46 (0.72 - 8.44)	0.15	2.38 (0.69 - 8.19)	0.17		
	Pathological Grade 5 vs. 1	2.73 (0.81 - 9.18)	0.11	2.68 (0.79 - 9.06)	0.11		
	Pathological Stage T3a vs. T2	1.14 (0.73 - 1.80)	0.56	1.14 (0.73 - 1.80)	0.56		
	Pathological Stage T3b vs. T2	1.82 (1.15 - 2.87)	0.01*	1.82 (1.15 - 2.86)	0.01*		
	Pathological Stage T3NOS vs. T2	1.36 (0.68 - 2.74)	0.38	1.35 (0.67 - 2.71)	0.40		
	Pathological Stage T4 vs. T2	2.93 (1.29 - 6.66)	0.01*	3.08 (1.36 - 6.98)	0.007*		
	LNI	1.61 (1.14 - 2.28)	0.008*	1.63 (1.15 - 2.31)	0.006*		

Odds ratios of genomic classifiers are per 0.1 unit increase. * indicates p-value < 0.05. [a]Bonferroni-Holm adjusted p-values.

Supplementary Figures

Supplementary Figure 1: Patient demographic information in test ordered and unordered populations.

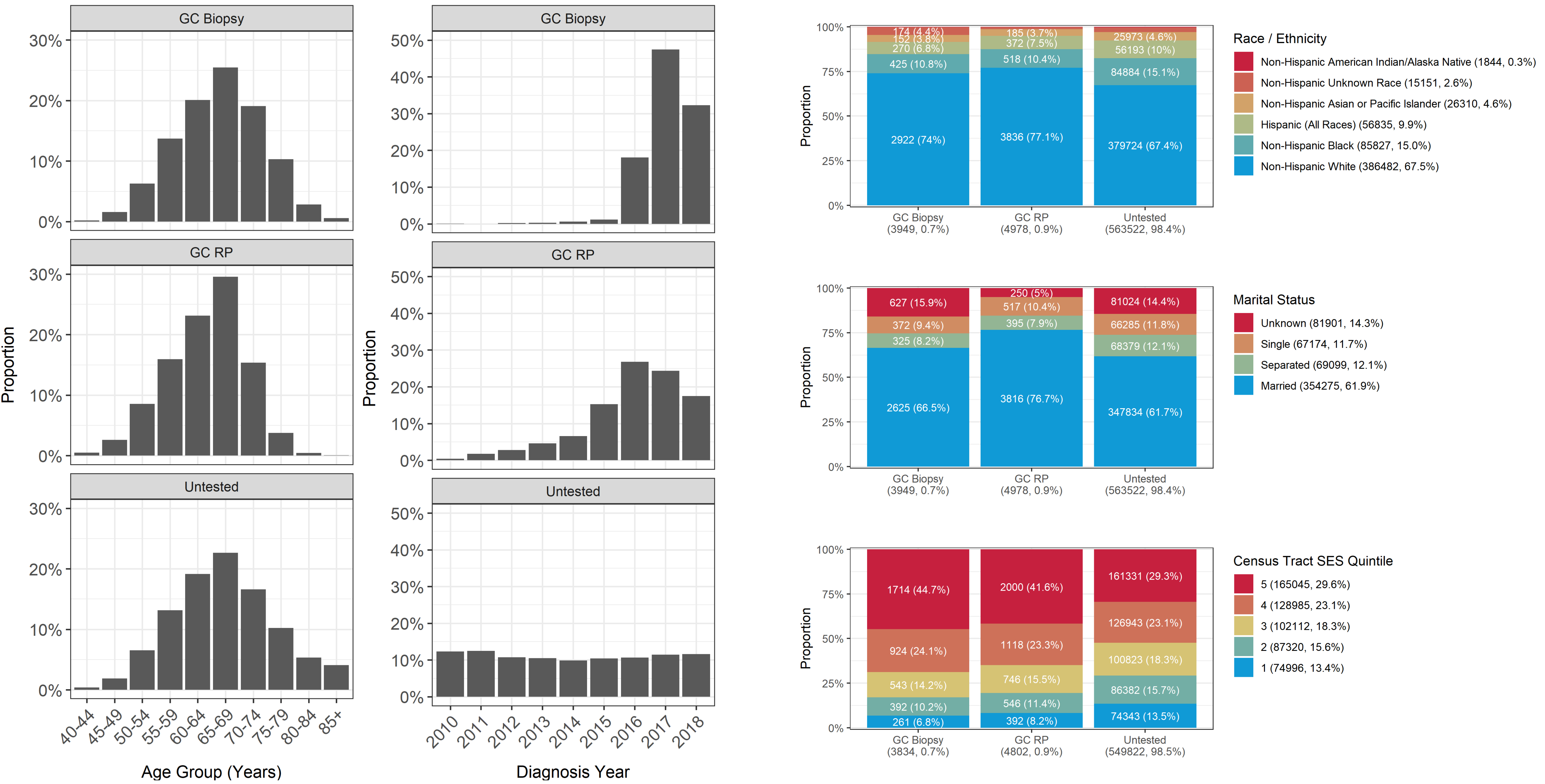

Supplementary Figure 2A: Plots of GC biopsy scores by clinicopathologic features.

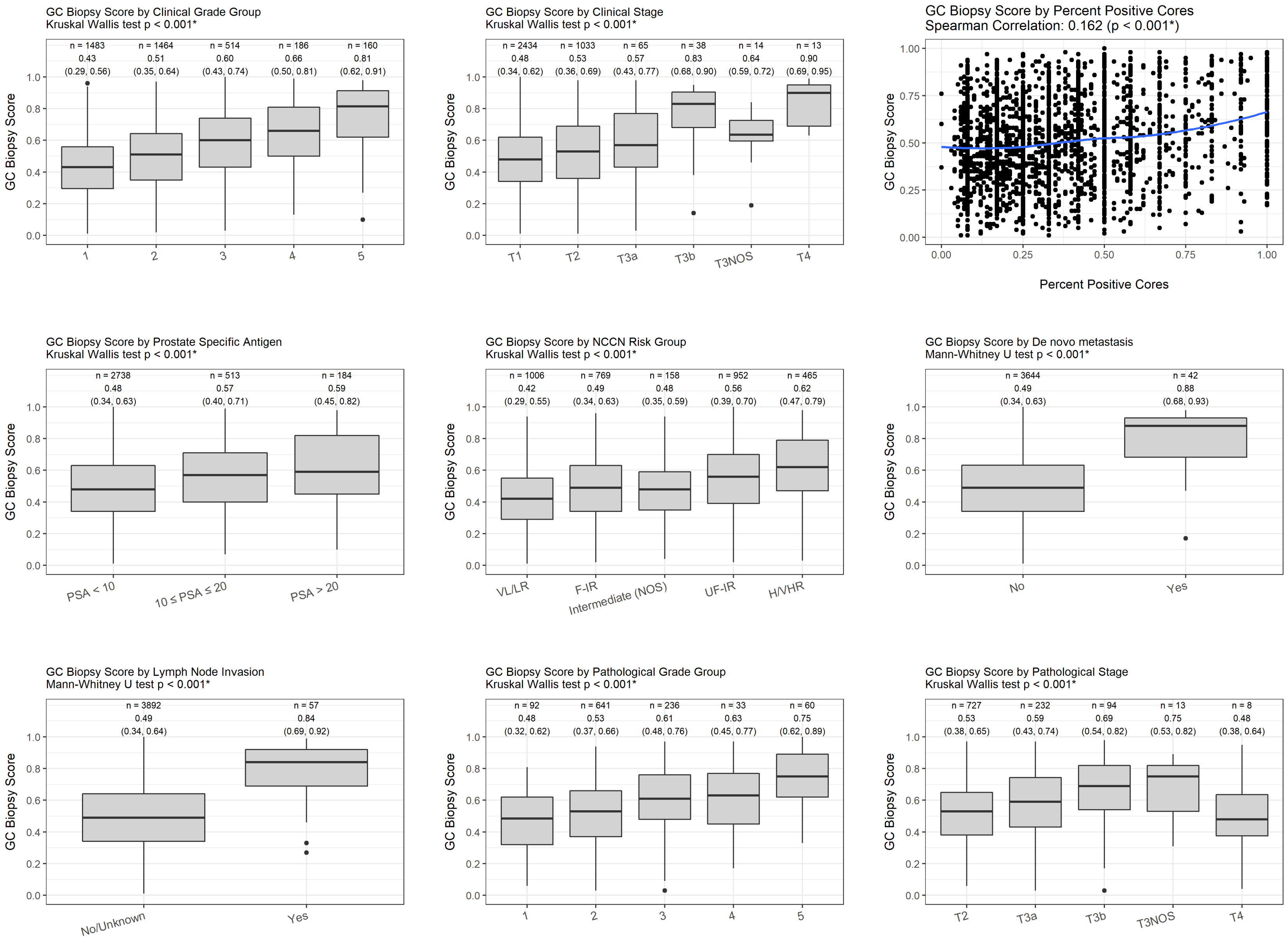

Supplementary Figure 2B: Plots of GC RP scores by clinicopathologic features.

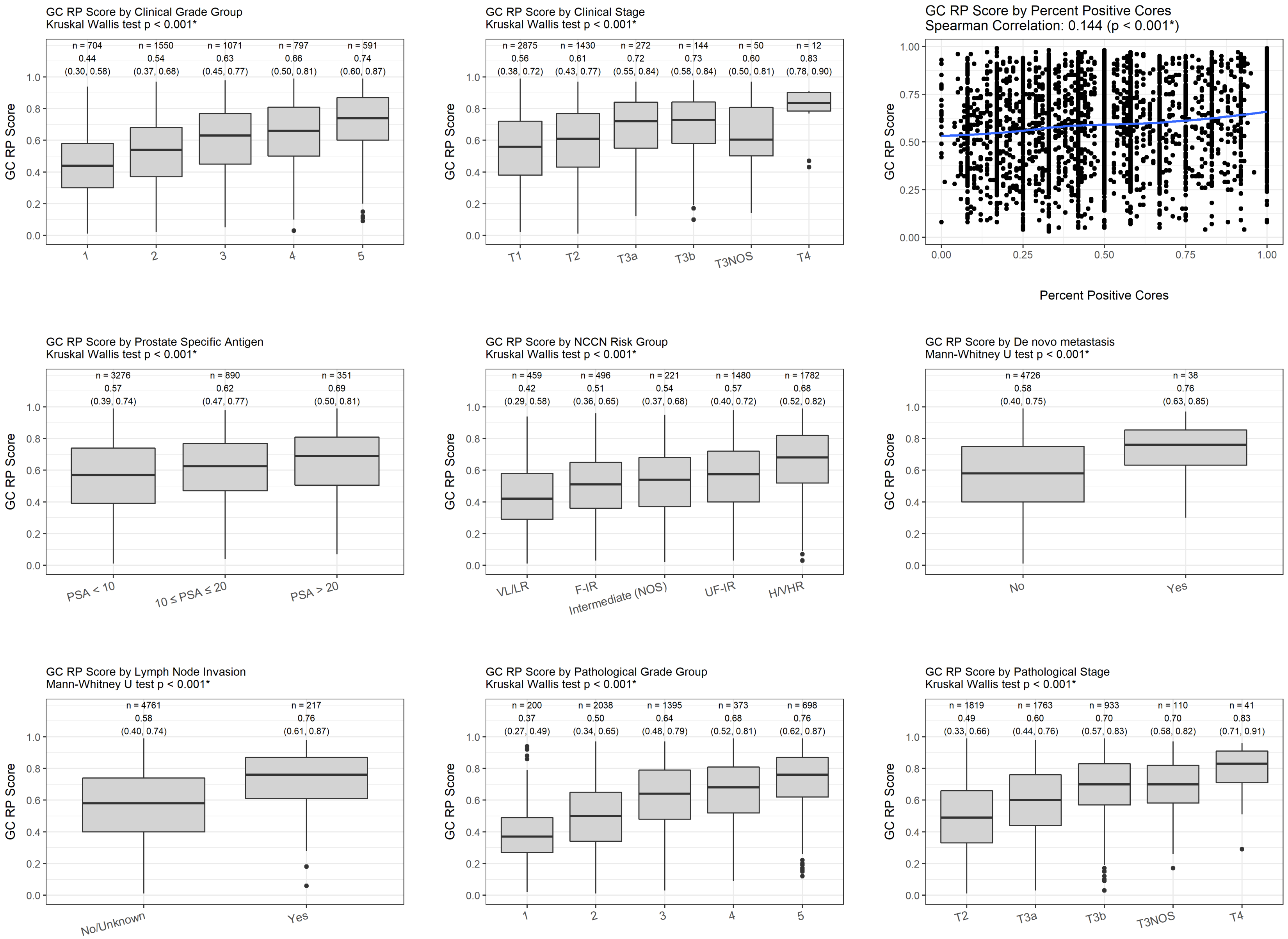

Supplement: pkad052_Supplementary_Data [file pkad052_supplementary_data.pdf]
